# Identification of Novel RNA Binding Proteins Influencing Circular RNA Expression in Hepatocellular Carcinoma

**DOI:** 10.3390/ijms22147477

**Published:** 2021-07-12

**Authors:** Rok Razpotnik, Petra Nassib, Tanja Kunej, Damjana Rozman, Tadeja Režen

**Affiliations:** 1 Centre for Functional Genomics and Bio-Chips, Faculty of Medicine, Institute of Biochemistry and Molecular Genetics, University of Ljubljana, 1000 Ljubljana, Slovenia; rok.razpotnik@mf.uni-lj.si (R.R.); petra.ivanusa@mf.uni-lj.si (P.N.); damjana.rozman@mf.uni-lj.si (D.R.); 2 Department of Animal Science, Biotechnical Faculty, University of Ljubljana, 1230 Domžale, Slovenia; tanja.kunej@bf.uni-lj.si

**Keywords:** circular RNA (circRNA), hepatocellular carcinoma (HCC), RNA binding proteins (RBPs), ESRP2

## Abstract

Circular RNAs (circRNAs) are increasingly recognized as having a role in cancer development. Their expression is modified in numerous cancers, including hepatocellular carcinoma (HCC); however, little is known about the mechanisms of their regulation. The aim of this study was to identify regulators of circRNAome expression in HCC. Using publicly available datasets, we identified RNA binding proteins (RBPs) with enriched motifs around the splice sites of differentially expressed circRNAs in HCC. We confirmed the binding of some of the candidate RBPs using ChIP-seq and eCLIP datasets in the ENCODE database. Several of the identified RBPs were found to be differentially expressed in HCC and/or correlated with the overall survival of HCC patients. According to our bioinformatics analyses and published evidence, we propose that NONO, PCPB2, PCPB1, ESRP2, and HNRNPK are candidate regulators of circRNA expression in HCC. We confirmed that the knocking down the epithelial splicing regulatory protein 2 (ESRP2), known to be involved in the maintenance of the adult liver phenotype, significantly changed the expression of candidate circRNAs in a model HCC cell line. By understanding the systemic changes in transcriptome splicing, we can identify new proteins involved in the molecular pathways leading to HCC development and progression.

## 1. Introduction

Circular RNAs (circRNAs) are a newly identified class of RNA molecules that have various roles, such as sponging miRNAs and RNA binding proteins (RBPs), modulating RBP function, having protein-coding potential, and regulating gene expression. [1]. They have already been implicated in various physiological and pathological processes, especially in cancer, where deregulated circRNAs can act as potential oncogenes or tumor suppressors, including in hepatocellular carcinoma (HCC) [2,3]. The tissue-specific expression, enrichment in extracellular vesicles, and increased stability of circRNAs indicate their potential as new biomarkers [4]. The differential expression of circRNAs has been studied in different cancer pathologies; however, what drives the differential expression of circRNAome remains largely unknown.

Though the biogenesis of circRNAs is a well-described mechanism, the regulation of their expression is not yet fully understood. The best-described mechanism of circRNA biogenesis is through the pairing of orientation-opposite complementary sequences (especially Alu repetitive elements) in the introns flanking the circRNA sequence, thereby bringing back-splice splicing sites to proximity. The accumulation of short interspersed nuclear repetitive DNA elements (SINEs) also positively correlates with increased circRNA variety and number in more complex organisms [5]. Other mechanisms, such as the presence of long flanking introns, have been shown to drive the biogenesis of a class of circRNAs [6]. Different studies have shown that circRNAs are not just passive by-products of canonical splicing and that their expression is actively regulated [7,8,9,10]. The circRNA/mRNA expression ratio from the same gene locus can shift in different biological conditions since linear and circular transcripts can change their expression in different directions [8,9,10]. Core spliceosomal components have been shown to regulate circRNA expression [11], as well as different RBPs and splicing factors [6,12,13,14,15,16,17,18,19,20]. Quaking (QKI) is an example of a splicing factor that actively regulates circRNA biogenesis during the epithelial–mesenchymal transition [13].

HCC is the most common type of primary liver cancer. The incidence of liver cancer is increasing worldwide, and liver cancer was found to be the second most common cancer-related cause of death in men and the third by total deaths worldwide [21]. It is a deadly disease mainly because there are limited diagnostic, prognostic, and therapeutic approaches; therefore, new therapeutic targets and biomarkers are needed [22]. Though the differential expression and identification of novel oncogenic and tumor-suppressor circRNAs have been described in the context of HCC [2,3,23], the active regulators of their differential expression remain unknown. The differential and aberrant splicing and the differential expression of splicing factors have been identified in different liver pathologies [24,25]. Additionally, changes in splicing patterns are prevalent in HCC, not only for protein-coding genes but also non-coding genes [26].

**Figure 1:**
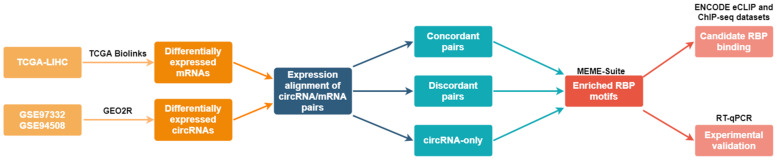
Schematic representation of the bioinformatical and experimental workflow for the identification of new regulators of circRNA expression in HCC.

The aim of this study was to identify new RBPs that regulate the differential expression of the circRNAome in HCC. By implementing our systems approach and using publicly available datasets (Figure 1), we discovered new RBPs with enriched motifs around the splice sites of differentially expressed circRNAs in HCC. We confirmed the binding of some RBPs upstream of the circRNA splice sites in the eCLIP and ChIP-seq ENCODE datasets. We knocked down the expression of an identified RBP, epithelial splicing regulatory protein 2 (ESRP2), in a model HCC cell line, and we showed that this modulated the expression levels of candidate circRNAs. Furthermore, we propose that ESRP2 is one of the RBPs driving the differential expression of circRNAs in HCC. Our study implemented a new approach for the identification of new RBPs regulating circRNA expression in HCC.

## 2. Results

### 2.1. Differentially Expressed circRNAs and mRNAs in HCC

**Figure 2:**
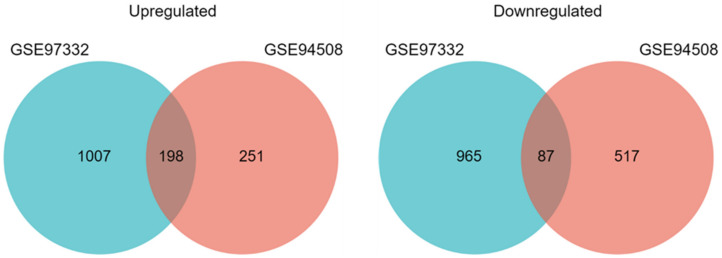
Identification of differentially expressed circRNAs in HCC. Venn diagrams of upregulated and downregulated circRNAs identified in the GSE97332 and GSE94508 datasets.

Two microarray datasets (GSE94508 [27] and GSE97332 [28]) were used to identify differentially expressed circRNAs in HCC. Both studies compared circRNA expression in tumor samples vs. adjacent normal tissue samples in five patients (GSE94508) and seven patients (GSE97332). The datasets originated from the same platform: Agilent-069978 Arraystar Human CircRNA microarray V1. We analyzed differentially expressed circRNAs using the GEO2R tool and identified 449 upregulated and 604 downregulated circRNAs in the GSE94508 dataset, and 1205 upregulated and 1052 downregulated circRNAs in the GSE97332 dataset. Subsequently, we intersected results from both datasets to identify high-confidence, differentially expressed circRNAs and used them for further analyses. We identified 197 upregulated and 87 downregulated circRNAs (Figure 2). We also found 424 circRNAs with no change in expression in both datasets and used them as a control group.

The TCGA-LIHC dataset from the TCGA (The Cancer Genome Atlas) project [29] was used to identify differentially expressed mRNAs in HCC in 371 tumors and 50 adjacent normal tissue samples. Subsequently, we aligned differentially expressed circRNA and mRNA pairs transcribed from the same host gene and divided them into groups according to the direction of differential expression (Table 1). For upregulated circRNAs, we found 88 concordant pairs (upregulated circRNA and mRNA), 33 discordant pairs (upregulated circRNA and downregulated mRNA), and 66 cases present in the circRNA-only group (only the circRNAs were differentially expressed) (Supplementary Materials, Table S1). Among the downregulated circRNAs, we found 11 concordant pairs, 36 discordant pairs, and 39 cases in the circRNA-only group (Supplementary Materials, Table S2).

### 2.2. Identification of Enriched RBPs Motifs in the Groups of Differentially Expressed circRNAs

We hypothesized that the change in expression of circRNAs in HCC is actively regulated by different RBPs. Therefore, we searched for the enrichment of RBP motifs 1000 bp upstream and downstream of the splice sites of all upregulated and downregulated circRNAs and each group from Table 1, separately. Using the AME tool in the MEME Suite software, we identified enriched motifs of RBPs around circRNA splice sites (Table 2; Supplementary Materials, Table S3). Enrichment was calculated over a control set of sequences (not differentially expressed circRNAs). The enrichment of motifs was more prominent in the groups of upregulated circRNAs, while, surprisingly, just a few motifs were found for groups of downregulated circRNAs. This suggests that the mechanisms driving the downregulation of circRNAs could be different from those driving the upregulation in HCC. The majority of enriched motifs corresponded to known splicing factors. Additionally, significantly more enriched motifs were found upstream than downstream of splice sites. When comparing enriched motifs between different groups of upregulated circRNAs (concordant, discordant pairs, and circRNA-only), we observed that identified motifs were uniquely enriched to each group, with the exception of motifs for PPRC1 and ESRP2, which were enriched in two distinct groups. This indicates that RBPs involved in the splicing regulation of circular and linear transcripts from the host gene can be different according to the direction and concordance of expression between them.

Next, we analyzed available ENCODE datasets to confirm the binding of identified RBPs to upstream sequences of upregulated circRNAs. We searched for the binding sites of RBPs in ChIP-seq datasets for the HepG2 cell line (HNRNPK, PCBP1, PCBP2, NONO, and SRSF9) and eCLIP datasets for the HepG2 cell line (HNRNPK, PCBP1, PCBP2, FXR2, SRSF9, and RBM5) and the K562 cell line (NONO). HepG2 is a hepatocellular model cell line, and K562 is a model leukemia cell line. Peaks found within 1000 bp upstream of circRNA splice sites were identified as positive signals. These were aligned with our in silico prediction of motif enrichments for RBPs upstream of the splice sites of upregulated circRNAs (Table 3). This provides supporting evidence for the binding of RBPs to the predicted sites upstream of splice sites for some of the candidate upregulated circRNAs on the RNA or DNA level.

**Figure 3:**
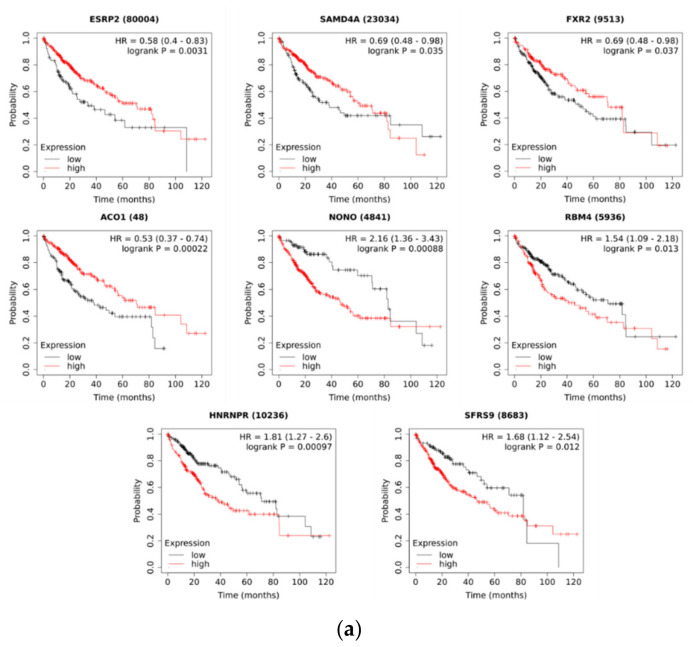

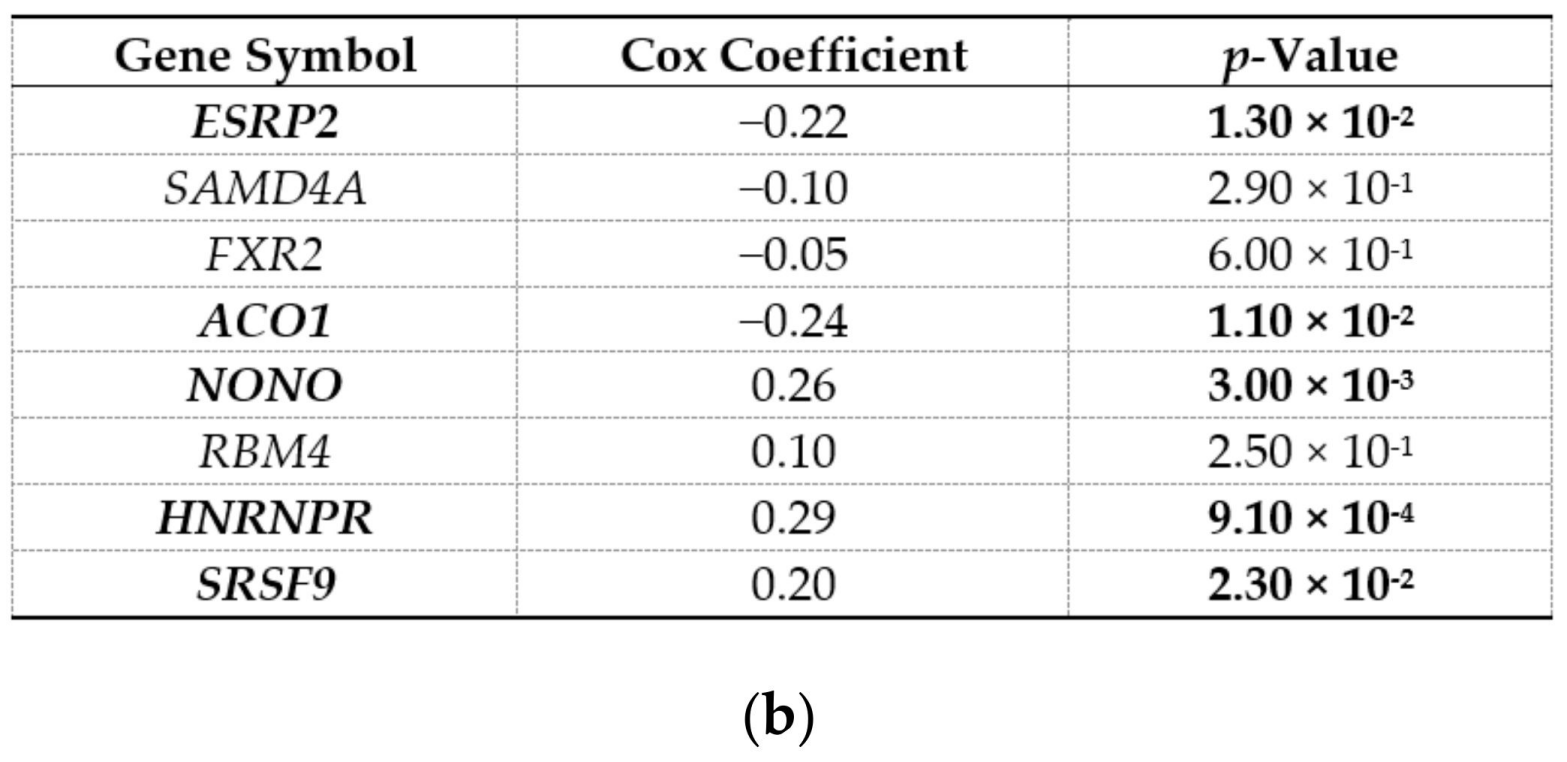
Correlation of RBP expression with the overall survival of HCC patients from the TCGA-LIHC dataset. (**a**) Kaplan–Meier survival analysis (identified by KM plotter). Hazard ratio (HR) and *p*-value are shown on individual plot. Low and high expression levels are drawn in black and red, respectively. (**b**) Multivariate Cox regression adjusted for age, gender, and grade (identified by OncoLnc). Bolded gene symbols denote significantly associated genes. Full gene names can be found in the footnote of Table 2.

We next hypothesized that RBPs with enriched motifs upstream of differentially expressed, upregulated circRNA sequences are also differentially expressed in HCC and that their expression correlated with patient survival. This would further confirm their potential for driving the splicing patterns in HCC. Indeed, the majority of the identified RBPs (17 out of 22) were differentially expressed in the TCGA-LIHC dataset (Table 4). Four of the differentially expressed RBPs were downregulated (*SAMD4A, ESRP2, ACO1,* and *FXR2*), while others were upregulated in HCC. Furthermore, the expression levels of some of the identified RBPs (8 out of 22) were associated with the overall survival of HCC patients from the TCGA-LIHC dataset (Figure 3a). In a multivariate Cox regression analysis adjusted for age, sex, and grade, five RBPs were still significantly associated with the survival of HCC patients (Figure 3b). We observed consistency between the direction of the differential expression of RBPs and the correlation with patient survival. For example, the expression levels of the four downregulated RBPs showed a positive correlation with the overall survival of HCC patients. The expression levels of four upregulated RBPs (*NONO, RBM4, HNRNPR,* and *SRSF9/SFRS9*) showed a negative correlation with the overall survival of HCC patients. These RBPs are therefore candidate regulators of circRNA expression with potential oncogenic or tumor-suppressor function in HCC.

### 2.3. ESRP2 Is a Potential Regulator of circRNA Expression in HCC

**Figure 4:**
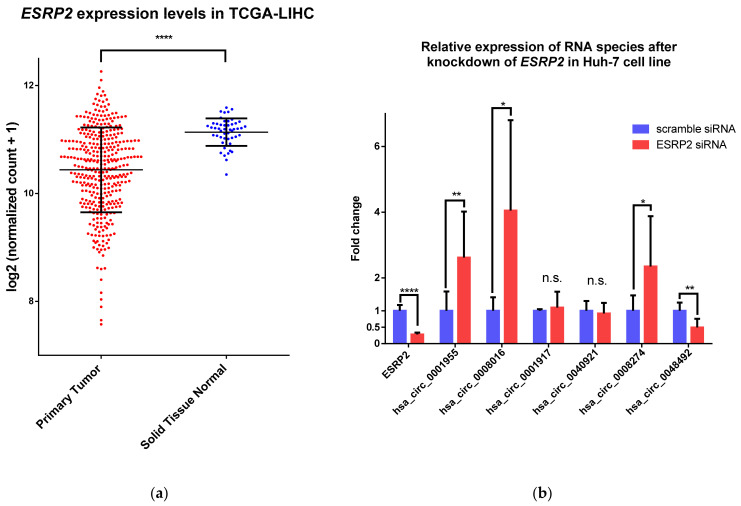
Downregulation of *ESRP2* expression in HCC tumors and the effect of this downregulation on circRNA expression. (**a**) *ESRP2* is significantly downregulated in HCC (TCGA-LIHC dataset); (**b**) The effect of the knockdown of *ESRP2* expression in Huh-7, an HCC model cell line, on the expression of the candidate circRNAs. Results from three independent biological triplicates are represented as averages, and error bars represent standard deviation. * *p*-value ≤ 0.05; ** *p*-value ≤ 0.01; **** *p*-value ≤ 0.0001; n.s.—not significant.

We next focus on ESRP2, since the motif recognized by ESRP2 was the most enriched for all upregulated circRNAs by the AME tool. However, no ENCODE eCLIP or ChIP-seq datasets for ESRP2 are currently available. ESRP2 has also not yet been linked to the regulation of circRNAs, but it has been linked to the regulation of alternative splicing changes in the process of liver maturation and the maintenance of the non-proliferative mature phenotype of adult hepatocytes [30]. First, we analyzed the expression of *ESRP2* in the TCGA-LIHC dataset, and we observed that it was downregulated (logFC = −1.54; adj. *p*-value = 1.53 × 10^−8^) in HCC (Table 4 and Figure 4a). Additionally, *ESRP2* expression levels were found to be positively correlated with the overall survival of HCC patients (Figure 3), which predicts the tumor-suppressor role of this RBP as the in the liver.

Next, we investigated whether the downregulation of *ESRP2* can influence the expression of upregulated circRNAs in HCC with the identified enriched ESRP2 motifs upstream of circRNA splice sites (Figure 4b). We confirmed that the knockdown of *ESRP2* expression in Huh-7, an HCC model cell line, upregulated the expression of hsa_circ_0001955, hsa_circ_0008016 and hsa_circ_0008274. This was expected since these circRNAs are upregulated in HCC, where *ESRP2* expression is downregulated. Interestingly, the expression of hsa_circ_0048492 was significantly downregulated upon the knockdown of *ESRP2* expression, which is contrary to what was observed in analyzed HCC datasets.

## 3. Discussion

There have been more than 400,000 circRNAs identified in human tissues; however, the roles of the majority of them have not yet been studied [31]. Though many of them are expressed at relatively low levels in cells, they have diverse molecular functions [1,32] and add another level of complexity to the regulation of cellular processes. The roles of circRNAs have already been elucidated in different pathologies, as well as in cancer; however, the regulation of their expression remains to be fully investigated. circRNA expression is proposed to be actively regulated, and the circular to linear transcript ratio from the same host gene can change in biological processes [8,9,10]. 

Our approach aimed at the identification of enriched motifs around the splice sites of differentially expressed circRNAs, and by taking this approach, we successfully identified new RBPs that could regulate circRNA expression in HCC. ENCODE datasets of eCLIP and ChIP-seq data enabled us to confirm the binding of some RBPs (NONO, PCBP1, PCPB2, and HNRNPK) to the in silico predicted enriched motifs upstream of upregulated circRNAs. Experimental evidence is already available for some of the RBPs identified in our study. For example, PCBP1, PCBP2, RBM4B, HNRNPK, and HNRNPH2 regulated circRNA expression from a vector expression cassette upon knocking down their expression in the human cell culture system [17]. RBM4 was also recognized as altering the circular-to-linear RNA expression ratio by using a minigene vector system in combination with exonic splicing enhancer sequences in the human cell culture system [33]. Furthermore, the epitranscriptomic nucleotide modification m6A is important for YTHDC1-mediated circRNA biogenesis [34]. Finally, a study using ENCODE datasets identified NONO as one of the most enriched RBPs in the flanking intron regions for a group of most highly expressed circRNAs in the K562 cell line [6]. 

Several RBPs identified in our study (HNRNPK, PCBP2, PCBP1, NONO, HNRNPH2, RBMX, and RBM8A) have already been shown to have an active role in HCC [35,36,37,38,39,40,41,42,43,44]. Our study confirms that the majority of these RBPs are differentially expressed in the TCGA-LIHC dataset and that their expression levels generally correlate to the overall survival of HCC patients. This is in line with our hypothesis that these RBPs are mediators of splicing changes in HCC and could influence the expression of circRNAome. According to our bioinformatics analyses and published evidence, we propose that NONO, PCPB2, PCPB1, and HNRNPK are strong candidates as regulators of circRNA expression in HCC. We also propose members of the RBM family, such as RBM4B, as new potential regulators of the splicing and expression of circRNAs in HCC.

Next, we focused our attention on ESRP2, an RBP and a splicing factor that is known to control and support the adult splicing program in the liver [30]. Its downregulation is part of the liver regeneration process [45], but it is also downregulated in alcoholic hepatitis [46] and HCC [47]. ESRP2 has not yet been linked to the regulation of circRNA biogenesis. Our analysis shows that its binding motif is most enriched in the upstream regions of upregulated circRNAs in HCC. Since its expression is downregulated in HCC and expression levels are positively correlated to the overall survival of the HCC patients, we hypothesized that ESRP2 can have an active role in the regulation of differentially expressed circRNAs in HCC. By knocking down the expression of *ESRP2* in the HCC model cell line Huh-7, we observed a significant upregulation of three circRNAs (hsa_circ_0001955, hsa_circ_0008016, and hsa_circ_0008274). These circRNAs are upregulated in HCC, and the oncogenic role in HCC was already confirmed for two [48,49]. Since these circRNAs are upregulated after *ESRP2* knockdown in cell lines, this indicates that ESRP2 could inhibit the biogenesis of these circRNAs in the liver. The active inhibition of circRNA expression by RBPs has been shown previously for ADAR1 [14], DHX9 [16], and HNRNPM [20]. It would be interesting to test in vivo whether ESRP2 also maintains a non-proliferative mature phenotype in the liver by regulating circRNA expression. The contradiction between the expression of hsa_circ_0048492 in our study and public data could be explained by the fact that the in vitro experimental conditions do not necessarily represent the complexity of the liver tissue, where additional factors besides ESRP2 may play a role in the regulation of its expression.

Our in silico analyses of the enrichment of RBP motifs revealed distinct RBP groups depending on the concordance in differential expression between pairs of circRNA–mRNA from the same host gene. This observation implicates the presence of different splicing mechanisms in the regulation of circRNA and linear RNA expression from the same host gene in HCC. The lack of significantly enriched RBP binding motifs in downregulated circRNAs was surprising, indicating a more complex interplay of cis- and trans-acting factors at these gene loci. For example, epigenetic factors, such as histone modifications and DNA methylation, have been associated with circRNA expression levels [50,51]. ADAR and DHX9 have been shown to actively repress the expression of circRNAs by binding to a double-stranded RNA or Alu elements [14,16]. Another discordance was in larger and more diverse enrichment in the upstream regions compared to the downstream regions of splice sites of upregulated circRNAs. This finding was supported by a recent study that identified a class of circRNAs with aberrant splicing that differs between upstream and downstream regions [6]. Another example of this phenomenon is ESRP2, for which it has been shown that the position of the binding site defines the fate of the exon [52,53]. If the binding site is in the upstream region, exon skipping is promoted, but if the binding site is in the downstream region, exon inclusion is promoted. Therefore, the position of binding sites seems to be relevant for circRNA biogenesis.

In our study, the concordance between circRNA and mRNA expression was analyzed using data collected by different technologies from unrelated patient cohorts, which made the alignment of expression more difficult. Therefore, we included intersected circRNAs from two studies in the bioinformatics analyses. Since our analysis identified RBPs that were also previously experimentally validated, we are confident that our novel approach to identify RBPs that actively regulate circRNA expression can be extrapolated to other research questions.

In conclusion, circRNAs add an additional layer of regulation in cancerogenesis, and deciphering the regulation of their expression will increase our understanding of the molecular pathways involved in HCC development. Our study identified new RBPs, which are strong candidates for regulators of circRNA expression in HCC. By understanding the systems changes in transcriptome splicing, we can identify new crucial players involved in the molecular mechanisms of HCC development and, consequently, identify new potential therapeutic targets.

## 4. Materials and Methods

### 4.1. Identification of Differentially Expressed circRNAs and mRNAs in HCC

The expression levels of circRNAs in HCC were obtained from the GEO database [54], where each microarray dataset of paired HCC and para-tumorous tissues (5 paired samples for GSE94508 [27] and 7 paired samples for GSE97332 [28]) was analyzed by GEO2R, an interactive tool used for analyzing the differential expression of GEO datasets (http://www.ncbi.nlm.nih.gov/geo/geo2r/, accessed on 17 April 2019). Log transformation was applied prior to the analysis, and *p*-values were adjusted for Benjamini and Hochberg false discovery rate (FDR). circRNAs with adjusted *p*-values ≤ 0.05 were marked as differentially expressed. There was no cut-off value for fold change in analyses. Venn diagrams were drawn using the draw.io software. 

The differential expression of mRNAs between liver cancer (371 cases) and adjacent normal tissue samples (50 cases) was analyzed in R using the TCGA-Biolinks package (accessed on 18 April 2019) [55], and *p*-values were adjusted for Benjamini and Hochberg FDR. mRNA with adjusted *p*-value ≤ 0.01 were marked as differentially expressed. There was no cut-off value for fold change in analyses. The visualization of the differential expression from the TCGA-LIHC cohort in the TCGA dataset was performed by GraphPad Prism 6 (GraphPad Software, San Diego, CA, USA).

### 4.2. Expression Alignment of circRNAs and Host Gene mRNAs

The Arraystar circRNA annotation of intersected differentially expressed and not differentially expressed circRNA candidates was converted to circBase annotation using supplementary tables from previous publications using Arraystar circRNA microarrays [56,57]. Though there are several annotations available for circRNAs throughout the literature, the circBase annotation [58] (http://www.circbase.org/, accessed on 15 May 2019) is most established and used. Two candidates with no known circBase alias were excluded from further study (1 candidate in upregulated and 1 candidate in not statistically significant group). circRNA genomic location, orientation, and host gene HGNC (HUGO Gene Nomenclature Committee) gene symbol were taken from circBase [58]. CircRNAs with antisense orientation relative to the host gene were excluded from further study (10 candidates in the upregulated group, 1 candidate in the downregulated group, and 6 candidates in the not statistically significant group). Change in expression was matched between circRNAs and host gene mRNAs by HGNC gene symbol. We split pairs of circRNA–mRNA from the host gene into three groups according to the direction of differential expression: pairs that changed expression in the same direction (both upregulated or downregulated) were marked as the group of concordant pairs, pairs that changed expression in the opposite direction were marked as the group of discordant pairs, and pairs where mRNA was not statistically significantly differentially expressed while the matched circRNA was were marked as the circRNA-only group. Intersected circRNAs that were not differentially expressed in both datasets (424 candidates) were used as a control set.

### 4.3. Identification of Enriched Motifs of RBPs in Groups of Differentially Expressed circRNAs

We searched for enriched motifs of RBPs around intersected, differentially expressed circRNA splice sites. The FASTA files of upstream and downstream sequences of circRNA splice sites (1000 bp) were obtained from circBase. Enriched motifs of RBPs in sequences around splice sites were identified in differentially expressed circRNAs using the Analysis of Motif Enrichment (AME), a tool in the MEME Suite software (https://meme-suite.org/meme/tools/ame, accessed on 17 May 2019) [59]. The motifs used for enrichment test were obtained from the CISBP-RNA database [60]. Fisher′s exact test was used as a motif enrichment test, and sequences from the control set of sequences (not differentially expressed circRNAs) were used as control sequences. Motifs with adjusted *p*-values ≤ 0.05 were considered to be significantly enriched in the tested groups. Enrichment was performed for all upregulated and downregulated circRNAs, as well as for distinct groups of interest (concordant pairs, discordant pairs, circRNA-only).

### 4.4. Identification of Known RBP Binding Sites from Published eCLIP and ChIP-seq Data

In vitro binding data of RBPs were obtained from ChIP-seq data (narrowPeak bigBed files of conservative IDR thresholded peaks) for the HepG2 cell line (ENCSR519QAA, ENCSR872EVQ, ENCSR945NSF, ENCSR624GNZ, and ENCSR476BQA) and eCLIP data (normalized narrowPeak bed files from two replicates) for the HepG2 cell line (ENCSR828ZID, ENCSR256CHX, ENCSR339FUY, ENCSR773KRC, ENCSR989VIY, ENCSR973HOJ, ENCSR773KRC, and ENCSR489ABS) and the K562 cell line (ENCSR861PAR) using the ENCODE Portal database (https://www.encodeproject.org/, accessed on 17 June 2019) [61,62]. Since RBPs can also bind DNA and execute their functions at the chromatin level, we also included ChIP-seq datasets alongside with eCLIP datasets from ENCODE [63]. Peaks from all available datasets were visualized using the Integrative Genomics Viewer (IGV) [64]. Genomic coordinates obtained from circBase were transformed from hg19 to hg38 by using the LiftOver tool (https://genome.ucsc.edu/cgi-bin/hgLiftOver, accessed on 14 June 2019) [65]. We identified peaks, for which enrichment was found by AME tool, within 1000 bp upstream of candidate circRNA splice sites.

### 4.5. Survival Analysis

Kaplan–Meier (KM) analysis was analyzed using KM plotter (https://kmplot.com/analysis/, accessed on 9 November 2020) [66]. Kaplan–Meier analysis was performed for overall survival on the TCGA-LIHC cohort. Patients in the TCGA-LIHC cohort were divided into high- and low-expression groups according to the median gene expression. Associations with log rank *p*-values ≤ 0.05 were marked as significant. The multivariate Cox regression analysis of the TCGA-LIHC cohort, adjusted for age, sex, and grade, was analyzed by utilizing the OncoLnc tool (http://www.oncolnc.org/, accessed on 18 June 2021) [67]. Associations with *p*-values ≤ 0.05 were marked as significant.

### 4.6. Cell Culture Huh-7 and Knockdown of ESRP2 Expression

The Huh-7 cell line (300156, CLS, Eppelheim, Germany) was cultured in Dulbecco’s Modified Eagle’s Media (DMEM) high glucose (D6429, Sigma Aldrich, St. Louis, MO, USA), supplemented with 10% FBS and 1% penicillin/streptomycin. Cells were cultured in a humidified chamber at a constant 37 °C and 5% O_2_. The knockdown of *ESRP2* expression was performed using PepMute siRNA Transfection Reagent (SL100566, SignaGen Laboratories, Rockville, MD, USA). Cells were seeded at a density of 200.000 cells/well on a 6-well plate, where they reached approximate 50% confluence 24 h after seeding. Cells were transfected using the protocol provided by the manufacturer with 30 nM concentration of siRNA concentration. The cell medium was changed 24 h after transfection with complete medium, and cells were cultured for an additional 48 h before RNA extraction. siRNA sequences used for silencing were: siRNA-ESRP2 5′-GACUUAAUCCUCCUAGUUUtt-3′ [68] and siRNA-negative 5′-AGGUAGUGUAAUCGCCUUGtt-3′ (Non Specific Control 47% GC, Eurofins Genomics, Ebersberg, Germany) (siMAX siRNA, Eurofins Genomics, Ebersberg, Germany). Silencing was performed in three independent biological triplicates.

### 4.7. RNA Isolation, Reverse Transcription and RT-qPCR

Total RNA was isolated by TRI reagent (T9424, Sigma Aldrich, St. Louis, MO, USA) using the protocol provided by the manufacturer, and 5 µg of total RNA was treated with DNase I (04716728001, Roche, Basel, Switzerland) and reverse-transcribed with random hexamers using Maxima reverse transcriptase (EP0742, Thermo Scientific, Waltham, MA, USA) using the protocol of the manufacturer. cDNA was mixed with LightCycler 480 SYBR Green 1 Master (04707516001, Roche, Basel, Switzerland) and appropriate primers (300 nM concentration; Supplementary Materials, Table S4). Touchdown RT-qPCR [69] was performed alongside with negative controls (no reverse transcriptase and water) using a modified temperature program: 95 °C × 5′ for one cycle; 95 °C × 20″ and 66 °C × 10″ for 4 cycles by decreasing the annealing temperature for 2 °C per cycle; and 95 °C × 10″, 60 °C × 10″, and and 72 °C × 10″, followed by a read, for 45 cycles. The reactions were carried on the LightCycler 480 II instrument (Roche, Basel, Switzerland) and were run in technical triplicates. The obtained Ct values were normalized to the geometrical mean of beta-actin (*ACTB*) and 60S acidic ribosomal protein P0 (*RPLP0*), and the relative fold-change was determined using the 2-ΔΔCt method. Complete reactions from RT-qPCR were run and visualized on 1% agarose gel electrophoresis to confirm the predicted size of amplified products. Student′s two-tailed t-test was used for the statistical analyses of three independent biological triplicates, and *p*-values ≤ 0.05 were considered to be statistically significant. Results were visualized in GraphPad Prism 6 (GraphPad Software, San Diego, CA, USA). 

## Figures and Tables

**Table 1:** Expression alignment of differentially expressed circRNAs and mRNAs from the same host gene. ↑—upregulated; ↓—downregulated; x—not differentially expressed.

circRNA	mRNA	Expression Alignment	Number of Pairs
↑	↑	Concordant pairs	88
↑	↓	Discordant pairs	33
↑	x	circRNA-only	66
↓	↓	Concordant pairs	11
↓	↑	Discordant pairs	36
↓	x	circRNA-only	39

**Table 2:** Enriched motifs for RBPs in different groups of upregulated and downregulated circRNAs. All motifs were significantly enriched by an adjusted *p*-value ≤ 0.05. *—identified motifs from another organism; x—no significant motif for RBP was identified.

All		Concordant Pairs		Discordant Pairs		circRNA-Only	
Gene Sym.	Adj. *p*-Value	Gene Sym.	Adj. *p*-Value	Gene Sym.	Adj. *p*-Value	Gene Sym.	Adj. *p*-Value
**Upregulated circRNAs, upstream of splice site**							
*ESRP2*	1.32 × 10^−4^	*ESRP2*	5.78 × 10^−3^	*RBM8A*	6.82 × 10^−3^	*NONO*	9.28 × 10^−6^
*NONO*	2.67 × 10^−4^	*PPRC1*	4.54 × 10^−2^	*PPRC1*	3.12 × 10^−2^	*HNRNPK*	2.89 × 10^−4^
*PCBP2*	3.61 × 10^−4^	*RBM6*	4.69 × 10^−2^	*RBM4B*	3.76 × 10^−2^	SRSF4 *	1.19 × 10^−3^
*PPRC1*	9.13 × 10^−4^			*RBM4*	4.52 × 10^−2^	*PCBP1*	2.42 × 10^−3^
*YTHDC1*	1.03 × 10^−2^			*SAMD4A*	4.96 × 10^−2^	*RBMX*	1.39 × 10^−2^
*RBM4*	1.22 × 10^−2^					*HNRNPH2*	1.54 × 10^−2^
*RBMX*	1.48 × 10^−2^					*FXR2*	1.87 × 10^−2^
*ACO1*	1.64 × 10^−2^					*ESRP2*	2.39 × 10^−2^
*RBM5*	1.81 × 10^−2^					*PCBP2*	2.49 × 10^−2^
*RBM4B*	2.45 × 10^−2^					*SRSF9*	3.67 × 10^−2^
*HNRNPH2*	2.84 × 10^−2^						
SRSF4 *	3.69 × 10^−2^						
**Upregulated circRNAs, downstream of splice site**							
*RBMX*	9.51 × 10^−3^	x	x	*RBM4B*	2.90 × 10^−2^	x	x
*HNRNPK*	2.41 × 10^−2^						
*SAMD4A*	3.84 × 10^−2^						
**Downregulated circRNAs, upstream of splice site**							
x	x	*EIF4B*	6.26 × 10^−3^	x	x	x	x
**Downregulated circRNAs, downstream of splice site**							
HNRNPR *	9.20 × 10^−3^	x	x	x	x	*PABPC5*	1.89 × 10^−2^

* Gene names: *ESRP2*—epithelial splicing regulatory protein 2; *NONO*—non-POU domain-containing octamer binding; *PCBP2*—poly(rC) binding protein 2; *PPRC1*—PPARG related coactivator 1; *YTHDC1*—YTH domain containing 1; *RBM4*—RNA binding motif protein 4; *RBMX*—RNA-binding motif protein X-linked; *ACO1*—aconitase 1; *RBM5*—RNA binding motif protein 5; *RBM4B*—RNA binding motif protein 4B; *HNRNPH2*—heterogeneous nuclear ribonucleoprotein H2; *SRSF4*—serine and arginine rich splicing factor 4; *RBM6*—RNA binding motif protein 6; *RBM8A*—RNA binding motif protein 8A; *SAMD4A*—sterile alpha motif domain containing protein 4A; *HNRNPK*—heterogeneous nuclear ribonucleoprotein K; *PCBP1*—poly(rC) binding protein 1; *FXR2*—FMR1 autosomal homolog 2; *SRSF9*—serine and arginine rich splicing factor 9; *EIF4B*—eukaryotic translation initiation factor 4B; *HNRNPR*—heterogeneous nuclear ribonucleoprotein R; *PABPC5*—poly(A) binding protein cytoplasmic.

**Table 3:** Identified peaks upstream of upregulated circRNA splicing sites identified from ENCODE datasets.

Gene Symbol	circRNA	Experiment	Experiment ID
*MSFD12*	hsa_circ_0048492	NONO eCLIP K562	ENCSR861PAR
*ANKRD11*	hsa_circ_0040921	NONO eCLIP K562	ENCSR861PAR
*FGFR1*	hsa_circ_0008016	PCBP2 eCLIP HepG2	ENCSR339FUY
*ZMIZ1*	hsa_circ_0092313	PCBP2 eCLIP HepG2	ENCSR339FUY
*DCAF8*	hsa_circ_0014879	HNRNPK eCLIP HepG2	ENCSR828ZID
*LIN52*	hsa_circ_0000554	PCBP1 ChIP-seq HepG2	ENCSR872EVQ
*DENND3*	hsa_circ_0001827	PCBP1 ChIP-seq HepG2	ENCSR872EVQ
*DENND3*	hsa_circ_0001828	PCBP1 ChIP-seq HepG2	ENCSR872EVQ
*DCAF8*	hsa_circ_0014879	PCBP1 ChIP-seq HepG2	ENCSR872EVQ
*LIN52*	hsa_circ_0000554	NONO ChIP-seq HepG2	ENCSR923UTX
*DENND3*	hsa_circ_0001827	NONO ChIP-seq HepG2	ENCSR923UTX
*DENND3*	hsa_circ_0001828	NONO ChIP-seq HepG2	ENCSR923UTX
*DENND3*	hsa_circ_0001827	HNRNPK ChIP-seq HepG2	ENCSR519QAA
*DENND3*	hsa_circ_0001828	HNRNPK ChIP-seq HepG2	ENCSR519QAA

Gene names: *MFSD12*—major facilitator superfamily domain containing 12; *ANKRD11*—ankyrin repeat domain 11; *FGFR1*—fibroblast growth factor receptor 1; *ZMIZ1*—zinc finger MIZ-type containing 1; *DCAF8*—DDB1 and CUL4 associated factor 8; *LIN52*—lin-52 DREAM MuvB core complex component; *DENND3*—DENN domain containing 3.

**Table 4:** RBPs whose motifs were found enriched in the flanking regions of differentially expressed circRNAs in HCC and are also differentially expressed in the TCGA-LIHC dataset. Fold-change and false discovery rate (FDR) are given for each candidate. Full gene names can be found in the footnote of Table 2.

Gene Symbol	Fold-Change	FDR
*SAMD4A*	0.62	2.26 × 10^−8^
*ESRP2*	0.65	1.53 × 10^−8^
*ACO1*	0.77	1.85 × 10^−3^
*FXR2*	0.82	4.89 × 10^−4^
*PCBP1*	1.20	2.24 × 10^−4^
*PCBP2*	1.20	1.25 × 10^−3^
*EIF4B*	1.22	4.01 × 10^−3^
*RBM4B*	1.27	3.40 × 10^−4^
*SFRS9*	1.29	9.36 × 10^−6^
*RBM5*	1.33	4.59 × 10^−8^
*RBM4*	1.39	8.15 × 10^−7^
*HNRNPK*	1.40	1.36 × 10^−12^
*RBM6*	1.45	8.67 × 10^−10^
*HNRNPR*	1.47	6.28 × 10^−14^
*RBM8A*	1.64	3.87 × 10^−15^
*RBMX*	1.71	3.40 × 10^−20^
*NONO*	1.76	2.83 × 10^−14^

## Data Availability

Not applicable.

## Supplementary Materials

The following are available online at https://www.mdpi.com/article/10.3390/ijms22147477/s1.
