# Supplementary material for: Identification of Novel RNA Binding Proteins Influencing Circular RNA Expression in Hepatocellular Carcinoma
Source: Int J Mol Sci. 2021 Jul 12;22(14):7477. doi: 10.3390/ijms22147477 (PMC8307310; doi:10.3390/ijms22147477)
Supplement: Supplementary file 1 [file ijms-22-07477-s001.zip › Supplementary Table S4.pdf]

Table S4. Primer sequences used for RT-qPCR.

Gene	Forward/Reverse	Sequence
ACTB	Forward	CCA ACC GCG AGA AGA TGA
ACTB	Reverse	CCA GAG GCG TAC AGG GAT AG
RPLP0	Forward	TGC ATC AGT ACC CCA TTC TAT CA
RPLP0	Reverse	AAG GTG TAA TCC GTC TCC ACA AGA
ESRP2	Forward	GCA GAG ACA CAA GCA CCA CAT G
ESRP2	Reverse	TTC CCG TGA CAA GAA ACG AGC C
hsa_circ_0048492	Forward	CAC TCA GGC ACC GTC TGC
hsa_circ_0048492	Reverse	CAG GGG CTG AAG ATG AAG GG
hsa_circ_0001955	Forward	TGG TGC ATC TGC AAT AAC TCG
hsa_circ_0001955	Reverse	ATT TCC CAC ATG GTC CAA AGT
hsa_circ_0040921	Forward	ATG ACC GCA TGA AGA TCG CA
hsa_circ_0040921	Reverse	GCT CGA TGC TGT GCT GTA GA
hsa_circ_0008274	Forward	AAA AGC TGC TGC CAG AAT TGT C
hsa_circ_0008274	Reverse	AGA GAC TTA ATG GCG ACT TGG T
hsa_circ_0008016	Forward	TAT GCT TGC GTA ACC AGC AGC
hsa_circ_0008016	Reverse	CCA GGG CTG GGC TGA AAC AT
hsa_circ_0001917	Forward	TCT CTG AAA GTG CCA AAG ACC T
hsa_circ_0001917	Reverse	GCC GAG TTT TCT TTT GAG GC
